# Supplementary material for: A Ratiometric Fiber Optic Sensor Based on CdTe QDs Functionalized with Glutathione and Mercaptopropionic Acid for On-Site Monitoring of Antibiotic Ciprofloxacin in Aquaculture Water
Source: Nanomaterials (Basel). 2022 Mar 1;12(5):829. doi: 10.3390/nano12050829 (PMC8912570; doi:10.3390/nano12050829)
Supplement: Supplementary file 1 [file nanomaterials-12-00829-s001.zip › nanomaterials-1527329-supplementary.pdf]

Article

A Ratiometric Fiber Optic Sensor Based on CdTe QDs Functionalized with Glutathione and Mercaptopropionic Acid for On-Site Monitoring of Antibiotic Ciprofloxacin in Aquaculture Water

Xiao-Lin Yuan [1], Xiao-Yi Wu [1,2], Miao He [1], Jia-Ping Lai [1,*], and Hui Sun [2,*]

[1] School of Chemistry, South China Normal University, Guangzhou 510006, China; 2018022153@m.scnu.edu.cn (X.-L.Y.); 2112004007@e.gzhu.edu.cn (X.-Y.W.); 2018022147@m.scnu.edu.cn (M.H.)
[2] College of Environmental Science & Engineering, Guangzhou University, Guangzhou 510006, China
* Correspondence: 20081223@m.scnu.edu.cn (J.-P.L.); esesunhui@gzhu.edu.cn (H.S.)

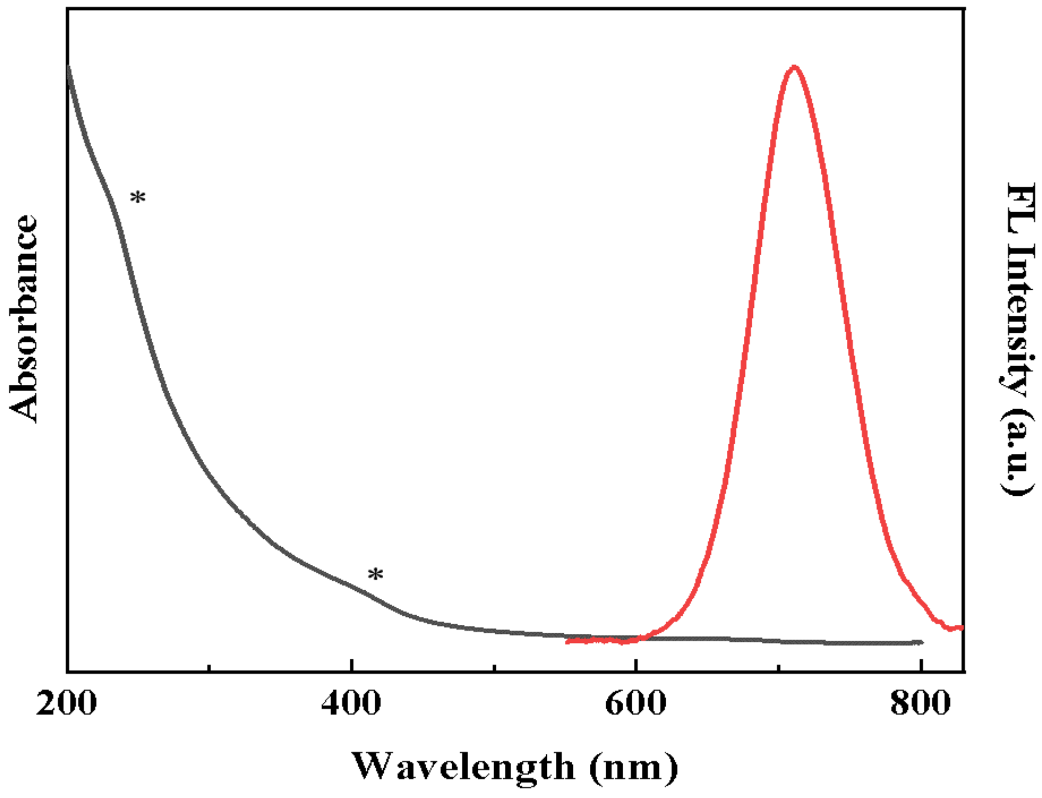

Figure S1. UV-absorption spectra and fluorescence emission spectra of GMPA@CdTe QDs.

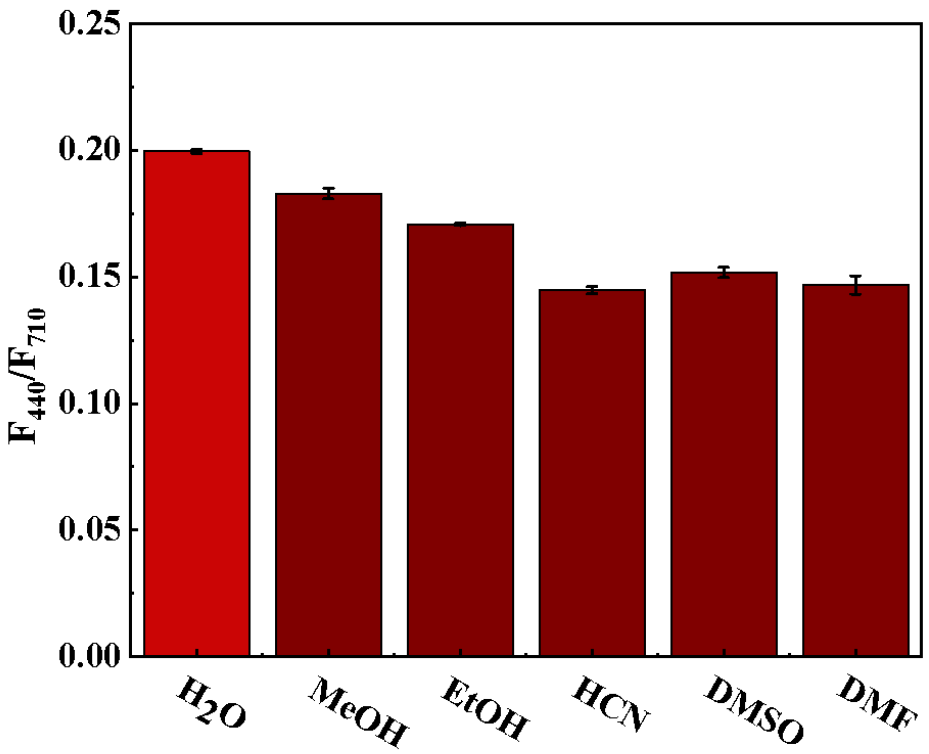

Figure S2. The influence of solvents on the signal of F440/F710.

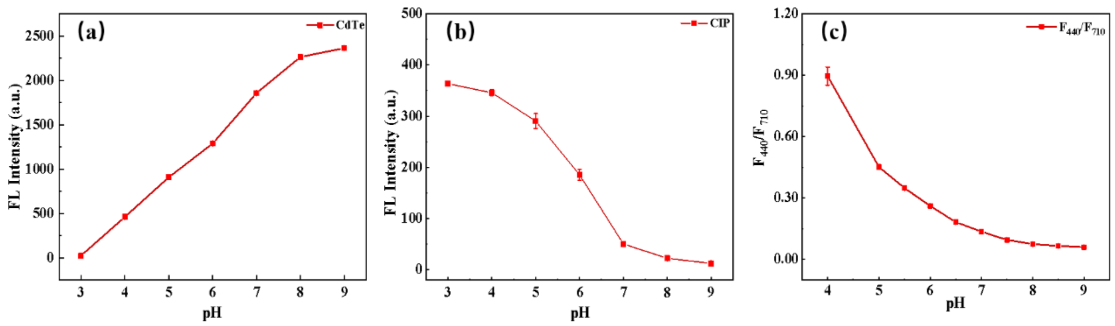

Figure S3. The influence of pH to the interaction between GMPA@CdTe QDs and CIP.

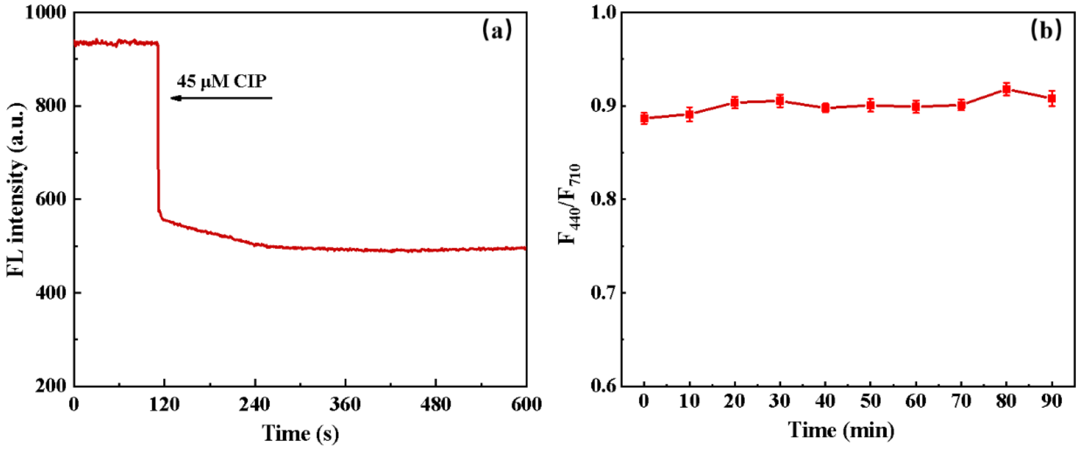

Figure S4. (**a**) The response time of the interaction between GMPA@CdTe QDs and CIP; (**b**) The stability of the fluorescence ratio (F440/F710) of GMPA@CdTe QDs in PBS.

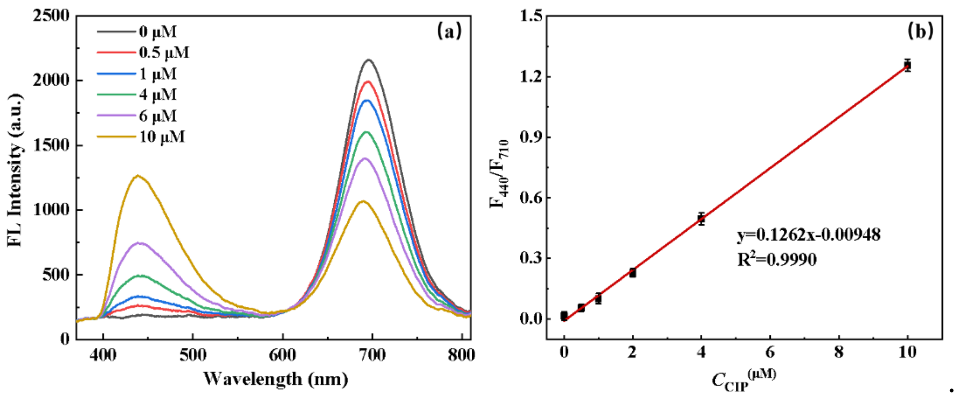

Figure S5. (**a**) Fluorescence spectra of the GMPA@CdTe QDs in the presence of various concentrations of CIP (0-10 mM); (**b**) The influence of CIP (0-10 mM) to the interaction between F440/F710 (FL-4600) (n=3).

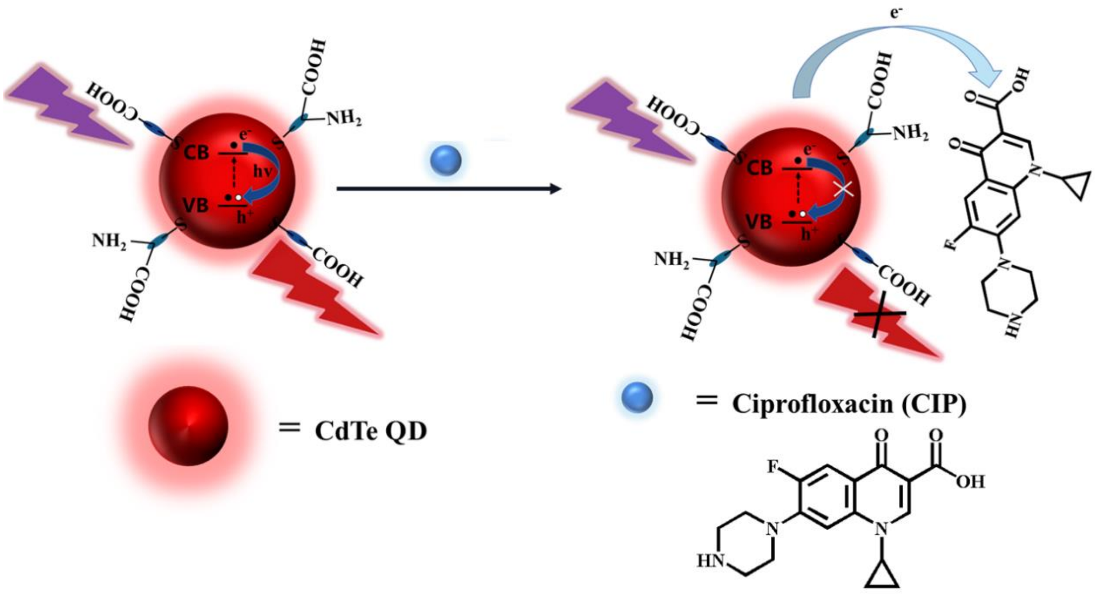

Figure S6. Schematic illustration of GMPA@CdTe QDs quenching by CIP.
